# A Small Natural Molecule Targeting IL-23/IL-17 Axis Exerts Dual Effects on T-Cell Function and Breast Cancer Cell Migration

**DOI:** 10.3390/medsci14040485

**Published:** 2026-08-15

**Authors:** Sara Bourdoukh, Khadija El Azhary, Sanaa Souat, Khouloud Ayed, Hamza Benthami, Said Byadi, Aziz Aboulmouhajir, Mohamed Elkarroumi, Asma Gati, Abdallah Badou

**Affiliations:** 1 Immuno-Genetics and Human Pathology Laboratory, Faculty of Medicine and Pharmacy, Hassan II University of Casablanca, Casablanca 20360, Morocco; sarabourdoukh@gmail.com (S.B.); elazhary.khadija6m@gmail.com (K.E.A.); sanaasouat2@gmail.com (S.S.); benthamihamza123@gmail.com (H.B.); 2 Laboratory of Genetics Immunology and Human Pathology, Faculty of Sciences of Tunis, University of Tunis El Manar, Tunis 2092, Tunisia; khouloud.ayed@etudiant-fst.utm.tn (K.A.); asma.gati@fst.utm.tn (A.G.); 3 Organic Synthesis, Extraction and Valorization Laboratory, Faculty of Sciences Ain Chock, Hassan II University of Casablanca, Casablanca 20360, Morocco; saidbyadii@gmail.com (S.B.); aboulmouhajir@gmail.com (A.A.); 4 Mohamed VI Oncology Center, Ibn Rochd University Hospital Center, Casablanca 20000, Morocco; elkarroumi7@hotmail.com; 5 City of Innovation and Technology Transfer, Hassan II University of Casablanca, Casablanca 20360, Morocco

**Keywords:** IL-23, Th17, IL-17A, IL-23RI, chronic inflammation, breast cancer, migration

## Abstract

**Background/Objectives:** Chronic inflammation in the tumor microenvironment (TME) promotes immune evasion and metastasis in breast cancer, where the IL-23/IL-17 axis is a key mediator. We investigated the expression of IL-23 in breast cancer and identified a small natural molecule therapeutically targeting this axis. **Methods:** IL-23 expression and its clinicopathological significance were assessed in a Moroccan breast cancer cohort and the METABRIC dataset. A High-throughput virtual screening of *Allium sativum* L. compounds was conducted to target the IL-23/IL-17 axis. In vitro, the selected candidate, IL-23RI, was evaluated for its effects on IL-17A and IFNγ production in human PBMCs using flow cytometry, as well as on the migration of MCF-7 and MDA-MB-231 breast cancer cells using wound-healing assays. **Results:** IL-23 was found to be overexpressed in aggressive breast cancer subtypes and correlated with unfavorable clinicopathological features and a poor prognosis. Elevated IL-23 expression was associated with an immunosuppressive TME, characterized by increased inhibitory immune checkpoints and immunosuppressive chemokines. In silico, IL-23RI demonstrates a high binding affinity for the IL-23 receptor, with a docking score of −7.482 kcal/mol and a binding free energy of −48.80 kcal/mol, stabilized by five hydrogen bonds. In vitro, IL-23RI selectively suppressed IL-17A production by CD4^+^ T cells without affecting IFNγ secretion by both CD4^+^ and CD8^+^ T cells. Furthermore, IL-23RI significantly inhibited the migration of MCF-7 and MDA-MB-231 breast cancer cells. **Conclusions:** These findings establish the IL-23/IL-17 axis as a critical therapeutic target and present IL-23RI as a promising dual-function agent for breast cancer treatment, concurrently modulating immunity and inhibiting tumor cell migration.

## 1. Introduction

Breast cancer represents a significant global burden as the leading cancer in women, with an estimated 2.31 million new cases and 665,684 deaths recorded in 2022, which corresponds to 6.9% of all cancer fatalities [1]. It exhibits a profound inter- and intra-tumoral heterogeneity, which is routinely classified in clinical practice into four main subtypes based on hormonal receptor profiles: Luminal A, Luminal B, HER2, and triple-negative breast cancer (TNBC). This molecular diversity is a critical determinant of therapeutic strategy and clinical prognosis [2,3]. The aggressiveness of TNBC and a subset of HER2-enriched tumors is driven by the frequent development of resistance to conventional therapies [4,5]. This resistance is mediated by complex interactions within the tumor microenvironment (TME) [6]. The crosstalk between immune and tumor cells is regulated by a complex cytokine network that drives chronic inflammation, inducing immunosuppression [7]. A central orchestrator of this pathogenic environment is the IL-23/IL-17 axis. In chronic inflammatory states, dendritic cells and macrophages serve as the main sources of interleukine-23 (IL-23). Together with other cytokines such as interleukine-1β (IL-1β), interleukine-6 (IL-6), and transforming growth factor-β (TGFβ), IL-23 drives the generation of pathogenic Th17 cells, which are characterized by their production of IL-17A [8]. In breast cancer, particularly in aggressive TNBC, high levels of IL-17A produced by Th17 cells correlate with a poor prognosis [9]. In fact, IL-17A promotes oncogenesis by inhibiting apoptosis, stimulating proliferation, angiogenesis, and facilitating metastasis via the upregulation of matrix metalloproteinase [10]. In aggressive breast cancer, IL-17A fosters chemoresistance through ERK1/2 signaling and enhances cell migration and invasion. Critically, these effects were abrogated by using an IL-17A-neutralizing antibody [11]. This underscores its significance as a key regulator in this pro-tumorigenic pathway. The differentiation and maintenance of these Th17 cells within the TME are conducted by a mixture of cytokines, including IL-1β, IL-6, TGF-β, and IL-23A. The latter acts by stabilizing the master transcription factor RAR-related orphan receptor gamma t (RORγt), which reinforces the Th17 cell’s immunosuppressive effector functions [12]. Beyond stabilizing the Th17 lineage, IL-23A enhances cancer cell proliferation and inhibits apoptosis through the JAK2/STAT3 signaling pathway while simultaneously sculpting an immunosuppressive TME [13]. This synergy between IL-23-driven inflammation and immune escape underpins the IL-23/IL-17 axis as a key driver of tumor progression.

The pivotal role of IL-23 in driving IL-17A secretion positions the IL-23/IL-17 axis as a compelling dual target for breast cancer therapy. Our team’s prior findings directly support this strategy, having demonstrated that a preparation from Moroccan *Allium sativum* L. significantly reduces IL-17A expression in human PBMCs [14]. This suggests the presence of a potent bioactive compound within the extract that probably interferes with the inflammatory IL-23/IL-17 axis.

This aligns with a major trend in immunotherapy; the advent of small-molecule inhibitors has represented a foundational pillar of cancer drug discovery [15]. These small inhibitors offer key advantages over monoclonal antibodies, including oral bioavailability, favorable pharmacokinetics, superior tumor tissue penetration and are associated with lower immunogenicity and production costs [16].

Therefore, this study was designed to confirm this innovative strategy. Our objectives are threefold: First, to delineate the expression profile and the potential functional role of IL-23 (IL-23A) within the breast tumor microenvironment. Subsequently, we will employ virtual screening to identify a specific natural small molecule from the Moroccan *Allium sativum* L., that can inhibit the interaction between IL-23 and its receptor IL-23R. Finally, we will evaluate the dual therapeutic potential of this novel small-molecule inhibitor, designated IL-23RI. We will investigate its capacity to modulate the immune response by suppressing IL-17A production through inhibition of the IL-23/IL-17axis, alongside assessing its anti-cancer activity against human breast cancer cells. This integrated approach aims to preliminarily establish IL-23RI as a promising immunotherapeutic candidate in breast cancer.

## 2. Materials and Methods

### 2.1. Patients and Samples

mRNA expression analysis was performed in 34 breast cancer patients with invasive breast carcinoma who underwent surgical treatment between 2019 and 2023. Tissue from breast reduction mammoplasty from healthy women served as a control. Freshly resected specimens were collected immediately after surgery at Mohamed VI Oncology Center, Ibn Rochd University hospital center (Casablanca, Morocco). Eligible patients were selected based on inclusion criteria, including a confirmed diagnosis of invasive breast carcinoma with defined molecular subtypes (Luminal A, Luminal B, HER2+, and TNBC) and provision of free and informed consent. However, the exclusion criteria were male sex, incomplete medical records and lack of free and informed consent. Clinicopathological data, such as age, grade, molecular subtype and biomarker status (estrogen receptor (ER), progesterone receptor (PR) and HER2 and Ki67) were defined by pathologists according to standard protocols and guidelines. These experimental procedures were approved by the Ethics Committee for Biomedical Research (CERB) of Ibn Rochd university hospital (approval code 28/15; approval date 20 October 2015).

### 2.2. METABRIC Dataset Analysis

Transcriptomic and clinicopathological data of 1904 primary invasive breast carcinomas were obtained from the Molecular Taxonomy of Breast Cancer International Consortium (METABRIC) cohort (METABRIC, Nature 2012 and Nat communication 2016) via the cBioPortal for Cancer Genomic interface (https://www.cbioportal.org/). The dataset was filtered to include only female patients with complete transcriptomic and clinicopathological data. Clinical annotation from the Data_clinical_patient.txt and data_clinical_sample.txt files was merged and mapped to their corresponding normalized log2 gene expression data (microarray). For survival prediction, patients were stratified into four distinct groups (excellent, good, moderate and poor) based on the Nottingham Prognostic Index (NPI) score. Statistical tests were independently repeated by two investigators to ensure result reliability.

### 2.3. Computational Analysis of TME

To assess the immunosuppressive state of the TME, expression data for inhibitory immune checkpoints and immunosuppressive chemokines were extracted from the METABRIC transcriptomic dataset. The cohort was then stratified into two patient groups according to IL-23A gene expression, using the median as the cutoff (IL-23A^low^ and IL-23A^high^). The correlation graphs were generated with RStudio software version (2025.05.0+496) and association analysis was performed using GraphPad prism 8.

### 2.4. Total RNA Isolation and Reverse Transcription

Total RNA was isolated from 34 fresh biopsies in addition to healthy control tissue using TRIzol reagent (Invitrogen, Massy, France), following the manufacturer’s instructions. RNA concentration and purity were quantified on a BioDrop µLite spectrophotometer (Fisher Scientific, Illkircg-Graffenstaden, France). For cDNA synthesis, 0.5 µg of total RNA was reverse-transcribed in a 20 µL reaction mixture containing 1 µL of Random Hexamer Primer (25 µg; Bioline, Livron-sur-Drôme, France), 0.5 µL of RNase inhibitor (Invitrogen, Massy, France), 0.5 µL of Tetro Reverse Transcriptase enzyme (Bioline, Livron-sur-Drôme, France), 4 µL of Tetro Reverse Transcriptase buffer (Bioline, France) and 4 µL of dNTPs (10 mM). The RNase-free water was added to adjust the final volume to 20 µL. The reaction mix was incubated at 25 °C for 10 min, followed by 42 °C for 45 min, and 70 °C for 15 min, and held at 4 °C.

### 2.5. Quantitative Real-Time PCR Assay

Gene expression was quantified using quantitative real-time PCR with the fluorescent dye SYBER^TM^ Green (Thermo Fisher Scientific, Waltham, MA, USA) on the croBEE^®^ real-time PCR detection system. Specific primer pairs for IL-23A and β-Actin were used at 10 µM in a final reaction volume of 20 µL. The reaction mix contained 2 µL of cDNA, 10 µL of SYBER^TM^ Green, 0.5 µL of each forward and reverse primer and 7 µL of nuclease-free water. A no-template control (NTC) was included in each run using 2 µL of nuclease-free water as a substitute for cDNA. The PCR cycling protocol comprised an initial hold at 95 °C for 10 min to activate polymerase and denature the samples, followed by 40 cycles of denaturation at 95 °C for 15 s and annealing/extension at 60 °C for 1 min. To confirm reaction specificity, a post-amplification melting curve analysis was performed. Relative quantification was computed using 2^−ΔΔCt^ approach. Data were examined as a relative mRNA expression using a housekeeping gene β-actin and healthy control tissue as internal control.

Primer pairs used:

- β-Actin Forward Primer: 5′-GAGATGGCCACGGCTGCTT-3′
- β-Actin Reverse Primer: 5′-GCCACAGGACTCCATGCCCA-3′
- IL-23A Forward Primer: 5′-CAGTGTGGAGATGGCTGTGA-3′
- IL-23A Reverse Primer: 5′-AGTAGGGAGGCATGAAGCTG-3′

### 2.6. High-Throughput Virtual Screening

#### 2.6.1. Protein Preparation

Protein preparation is a crucial step in molecular modeling and drug discovery that involves optimizing the structure of a protein molecule to ensure accuracy and reliability in docking analysis. Proper protein preparation enhances the quality and validity of computational results. The 3D crystal structure of human IL-23 Receptor (IL-23R) in complex with its ligand IL-23A (PDB ID: 5MZV) was downloaded from the RCSB Protein Data Bank (https://www.rcsb.org/). The protein structure was prepared for molecular docking using the Maestro Schrödinger Suite (2018-1) Protein Preparation Wizard. The preprocessing steps included removing atomic clashes, adding hydrogen atoms, optimizing hydrogen bonds, and assigning formal charges. Missing side chains were added, and then all heteroatoms and water molecules within the binding site were removed. The crystal structure was refined at neutral pH and subjected to energy minimization with the OPLS 2005 force field.

#### 2.6.2. Grid Generation and Preparation of Chemical Ligand Library

3D grid generation around the protein binding site is essential before docking, guiding ligand placement during docking simulations. This process integrates the protein’s structure and electrostatic properties, ensuring accurate ligand binding predictions. To define the receptor grid, the active sites of the protein (GLY24, ASN27, ASN29, THR 112, LEU113, ASP118 and GLU111) were obtained from structural data reported in a prior publication [17]. The grid was generated using default parameters, with a partial charge cutoff of 0.25 and a scaling factor of 1.0.

The chemical compounds derived from *Allium Sativum* L. were identified from published phytochemical studies (Supplementary Table S1) and downloaded in 3D SDF format from the PubChem database (https://pubchem.ncbi.nlm.nih.gov/). These compounds constituted the screening library. The LigPrep module within the Schrödinger Suite was used to prepare the ligands for virtual screening. Using the Epik application, ligands were generated in their various possible isomeric and tautomeric states at a physiological pH. All generated structures were optimized with the OPLS 2005 force field prior to high-throughput virtual screening.

#### 2.6.3. Virtual Screening

The virtual screening workflow tool within the Schrödinger Suite was employed for the molecular docking analysis. The prepared ligand file and the generated receptor grid file were selected as inputs. The docking protocol comprised three sequential phases: initial high-throughput virtual screening (HTVS), followed by Standard Precision (SP), and Extra Precision (XP) docking. The top-ranked poses from the XP stage were subsequently subjected to Molecular Mechanics-based Generalized Born Surface Area (MM-GBSA) calculations to predict binding free energies. The resulting outputs were analyzed to identify the compound exhibiting the highest binding affinity for IL-23R, as indicated by the most (negative) docking score.

### 2.7. PBMC Preparation and Cell Culture

Fresh venous blood was collected from healthy adult women volunteers at the Regional Blood Transfusion Center (Casablanca, Morocco) after obtaining written informed consent according to the regulations approved by the Ethics Committee for Biomedical Research of the faculty of medicine and pharmacy at Hassan II University (approval code 0216; approval date 3 March 2016). Peripheral blood mononuclear cells (PBMCs) were freshly isolated by density gradient centrifugation using Ficoll-histopaque (Sigma, Saint-Louis, MO, USA) at 1000 *g* for 20 min at 20 °C. PBMCs were then collected and washed twice with NaCl (0.9%) at 400 *g* for 10 min and resuspended in complete RPMI-1640 medium (Sigma, Saint-Louis, MO, USA), supplemented with 10% Fetal Bovine Serum (FBS) (Sigma, Saint-Louis, MO, USA), and 1% penicillin-streptomycin (Gibco, Life Technologies, Carlsbad, CA, USA). IL-23RI corresponds to a flavonoid derived from *Allium sativum* L. purchased from MedChemExpress (HY-15097R) (PubChem CID: 5281672; Purity ≥ 98% by HPLC). For the MTT assay, PBMCs were seeded into 96-well plates at a density of 1 × 10^5^ cells per well in triplicate with or without different doses of IL-23RI, and incubated for 72 h at 37 °C and 5% CO_2_ in a humidified atmosphere.

For flow cytometry analysis, PBMCs were seeded in 96-well plates at a density of 1 × 10^6^ cells per well in the presence or absence of IL-23RI treatment at 160 µM. These cells were activated with 2.5 µg/mL plate-bound human anti-CD3 (clone OKT3, GeneTex, Irvine, CA, USA) in complete RPMI medium in the presence or absence of a cytokine mix containing recombinant human IL-23 (50 ng/mL) (ABclonal Technology, Woburn, MA, USA) and IL-1β (50 ng/mL) (NKMAX Bio, Seongnam-si, Republic of Korea). For IL-17A inhibition analysis, cells were incubated at 37 °C, with 5% CO_2_ in a humidified atmosphere for 5 days, and for IFNγ analysis, cells were incubated overnight before staining.

### 2.8. Tumor Cell Line Culture

The human breast cancer cell lines MCF-7 (RRID:CVCL_0031) and MDA-MB-231 (RRID:CVCL_0062) were obtained from the American Type Cell Collection (ATCC, Manassas, VA, USA). The cells were cultured in complete Dulbecco’s modified Eagle medium (DMEM) (PAN Biotech, Aidebach, Germany) with L-Glutamine, supplemented with 10% FBS (PAN Biotech, Aidebach, Germany) and 1% penicillin and streptomycin (Gibco, Life Technologies, Carlsbad, CA, USA). For cell viability, MCF-7 and MDA-MB-231 were seeded into 96-well plates in triplicates at 8 × 10^3^ cells per well. After 24 h, the culture medium was replaced with various concentrations of IL-23RI for 24, 48 and 72 h at 37 °C, with 5% CO_2_ in a humidified atmosphere.

### 2.9. Wound-Healing Assay

MCF-7 and MDA-MB-231 cells were seeded in 6-well plates at a density of 4 × 10^5^ cells per well and grown to 80–90% confluence. In the middle of each well, a denuded zone was carefully created by scraping the cell monolayer with a 20 µL pipette tip. The detached cells were removed by washing with NaCl (0.9%), and breast cancer cells were incubated in complete DMEM containing various concentrations of IL-23RI for 24 and 48 h. Wound closure was monitored using an inverted microscope (Leica Microsystems, Wetzlar, Germany) at 0, 24 and 48 h. The area filled by migrating cells was quantified using ImageJ software, (version 1.52v), at each time point. Values are expressed as the mean ± SD of the migrated area relative to the initial scratch.

Wound closure (%) = 100 − ($\frac{At}{A0}\times 100$) where At is the wound area recorded at 24 or 48 h, and A0 is the wound area recorded at 0 h.

### 2.10. MTT Assay

Cell viability was assessed using an MTT (3-[4,5-dimethylthiazol-2-yl]-2,5 diphenyl tetrazolium bromide) assay. Cells were cultured and incubated as described above. Enzymatic activity was determined by MTT assay and compared to that of untreated cells. The cultural medium was discarded, and 100 µL of medium containing MTT (5 mg/mL, Sigma, Saint-Louis, MO, USA) was added to each well. Plate was incubated for 4 h at 37 °C, 5% CO_2_ in humidified atmosphere. The resulting formazan crystals were solubilized in 100 µL of dimethyl sulfoxide (DMSO). Subsequently, the optical density (OD) of each well was measured at 550 nm using a microplate reader (FLUOstar Omega, Ortenberg, Germany/SYNERGY LX, Winooski, VT, USA). Cell viability was calculated as a percentage of the absorbance measured in untreated control cells.

$$\mathrm{Cell}\;\mathrm{viability}\;(\%)=\frac{Mean\;of\;OD\;(treated\;cells)}{Mean\;of\;OD\;(untreated\;control\;cells)}\times 100$$

### 2.11. Flow Cytometry

Surface and intracellular markers were detected using the following FACS anti-human monoclonal antibodies: CD4-PE/Cy7 (Clone RFT4; SouthernBiotech, Birmingham, AL, USA); CD8-APC (Clone RPA-T8; MBL, Japan, Tokyo); IL-17A-PE (Clone eBio64DEC17; Invitrogen, Eugene, OR, USA) and IFNγ-FITC (Clone B27; ORIGENE, Rockville, MD, USA), and appropriate isotype controls. Brefeldin A (Invitrogen, Eugene, OR, USA) was added to cultured cells at a final concentration of 10 µg/mL for less than 12 h prior to staining. Then cell suspensions were washed and resuspended in staining buffer (phosphate-buffered saline (PBS) containing 2% FBS). For intracellular cytokine staining, cells were fixed with 4% Formaldehyde and permeabilized with 0.1% Tritton X-100. Samples were acquired using a BD FACSLyric^TM^ cytometer using BD FACSuite v 1.5 software. Photomultiplier tube (PMT) voltages were set using BD CS&T beads (BD Biosciences, San Jose, CA, USA). We set the statistical significance threshold at a *p*-value less than 0.05 for all tests.

### 2.12. Statistical Analysis

Statistical analysis and graphical representations were performed using GraphPad prism 8.0.1 and RStudio software version (2025.05.0+496). The Shapiro–Wilk normality test was employed to evaluate the distribution of the data. The Mann–Whitney rank test was used for assessing differences between two independent groups, while the Kruskal–Wallis test was applied for comparing multiple groups followed by Dunn’s multiple comparisons test. Spearman’s test was used for correlation coefficients. To define IL-23A gene expression status, the median was used as a cutoff to stratify our METABRIC cohort into IL-23A^low^ and IL-23A^high^ clusters. For normally distributed data, one-way ANOVA followed by Dunnett’s post hoc test was used. For non-normally distributed data, the Friedman test with Dunn’s post hoc test was applied for paired data. Biological replicates refer to independent experiments performed with different donors or different cell passages. Technical replicates refer to triplicates within the same experiment. For MTT assays, each condition was tested in triplicate within each experiment, and the assay was repeated three times independently (biological replicates). For the wound-healing assay, three independent experiments were performed (biological replicates). For IL-17A inhibition and IFNγ expression assays, PBMCs from independent healthy donors were used (*n* = 7 and *n* = 5, respectively).

### 2.13. Generative AI Statement

No generative AI tools were used in the preparation of this manuscript. The authors used Grammarly (version 1.2.279.1925) and Reverso (version 2.16.1.935) for language editing and grammar checking to improve readability.

## 3. Results

### 3.1. IL-23 Was Associated with Aggressive Clinical Features in Breast Cancer Patients

**Figure 1:**
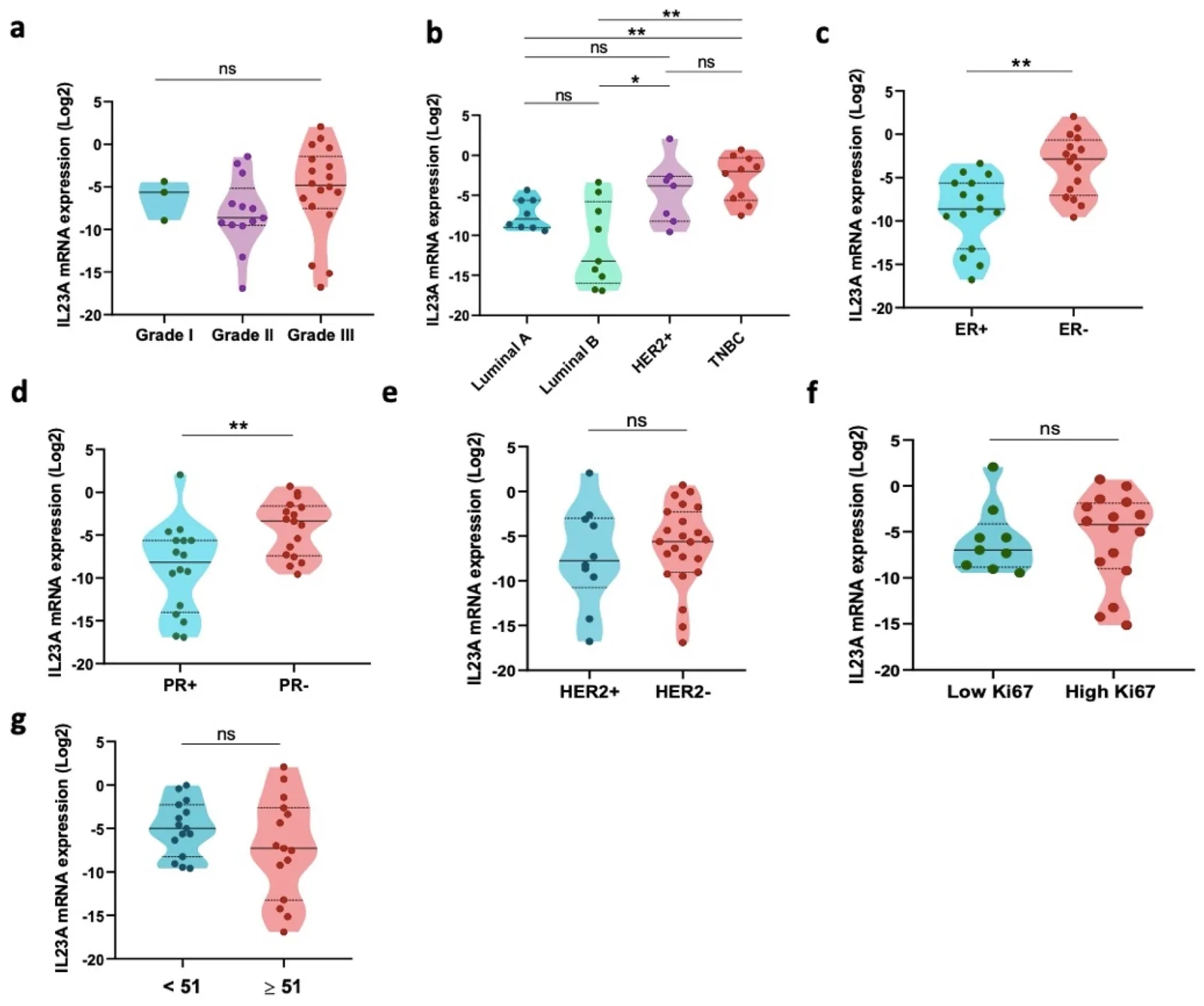
IL-23 transcript levels were linked to unfavorable clinicopathological features in Moroccan cohort. (**a**) IL-23 mRNA expression level in distinct tumor grade (I, II, III). (**b**) Analysis of mRNA expression levels of IL-23 among intrinsic molecular subtypes (Luminal A, Luminal B, HER2+, and TNBC). (**c**,**d**) IL-23 mRNA expression based on hormonal parameters (ER and PR). (**e**) IL-23 mRNA expression according to HER2 status. (**f**) IL-23 mRNA expression level stratified by Ki67 index. (**g**) IL-23 mRNA expression level relative to patients age. Significance was calculated using Mann–Whitney test. * *p* < 0.05, ** *p* < 0.01, and ‘ns’ indicates no significance.

To explore the expression pattern of IL-23 in human breast tumorigenesis, we first analyzed an in-house cohort of 34 Moroccan patients with invasive carcinoma. The clinicopathological parameters of the enrolled patients are summarized in Table 1. IL-23 (IL-23A) mRNA levels were quantified by RT-PCR from fresh tissue samples and represented as log2 (2^−ΔΔCt^). Our findings revealed that IL-23 transcript levels were not significantly associated with tumor grade (Grade II vs. Grade III: *p* = 0.0564) (Figure 1a), but interestingly, its expression was significantly elevated in HER2-positive tumors (*p* = 0.0311) and triple-negative breast cancer (TNBC) compared to luminal subtypes (TNBC vs. Luminal A: *p* = 0.0044, TNBC vs. Luminal B: *p* = 0.0021) (Figure 1b). Notably, IL-23 mRNA levels were markedly upregulated in tumors with negative Estrogen (*p* = 0.0011) and Progesterone (*p* = 0.0059) receptors (ER and PR) status compared to positive phenotypes (Figure 1c,d), while no significant correlation was observed with HER2 status (*p* = 0.5499) (Figure 1e). However, regarding the proliferation index Ki67 (*p* = 0.5594) (Figure 1f) and patient age (*p* = 0.3669) (Figure 1g), no significant association was observed with IL-23 expression levels.

**Figure 2:**
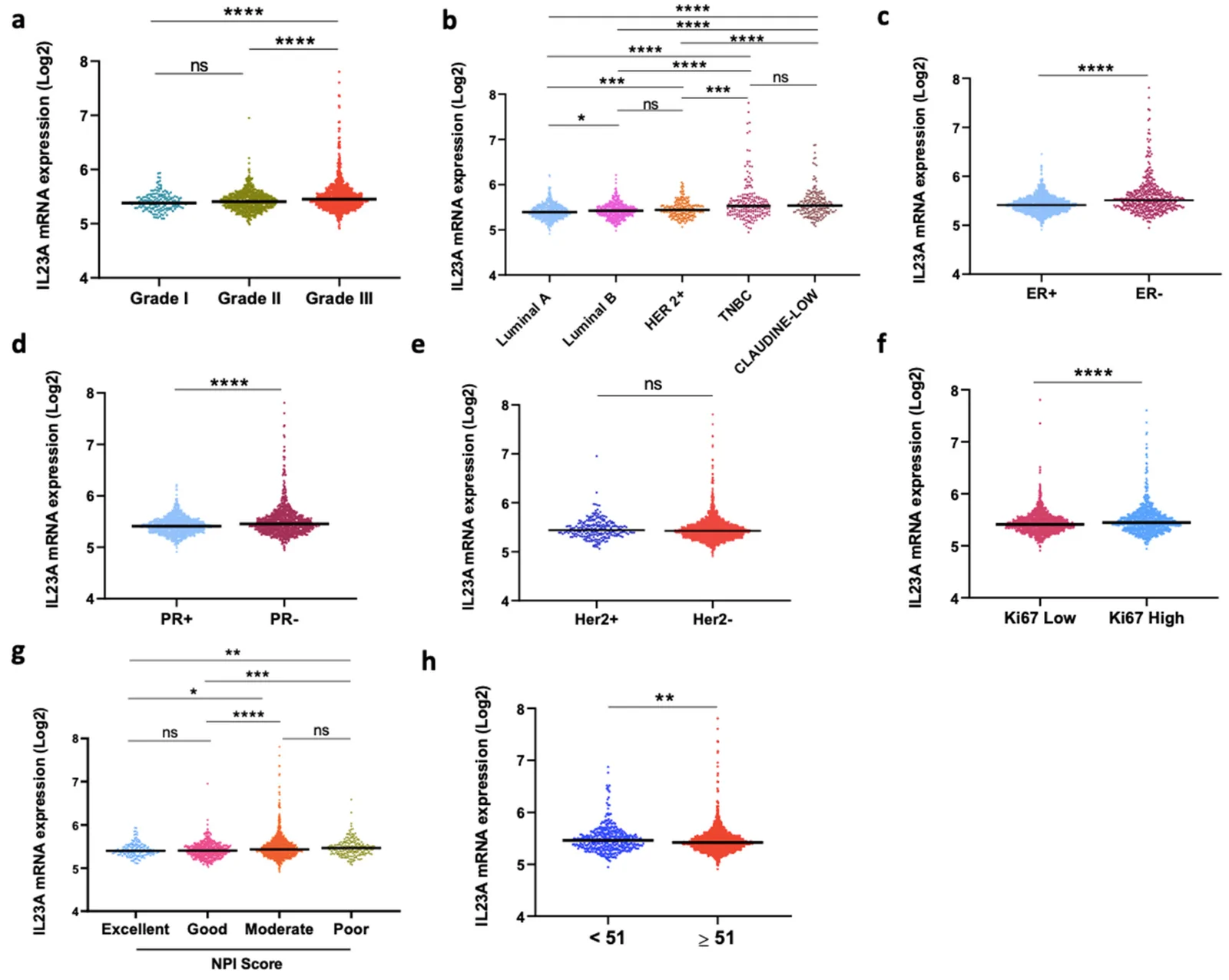
IL-23 gene expression was associated with aggressive clinical features in the METABRIC cohort. (**a**) Analysis of mRNA expression levels of IL-23A among intrinsic molecular subtypes (Luminal A, Luminal B, HER2+, and TNBC). (**b**,**c**) IL-23A mRNA expression based on hormonal parameters (ER and PR). (**d**) IL-23A mRNA expression according to HER2 status. (**e**) IL-23A mRNA expression level in distinct tumor grade (I, II, III). (**f**) IL-23 mRNA expression level stratified by Ki67 index. (**g**) IL-23 mRNA expression stratified by Nottingham Prognostic Index (NPI). (**h**) IL-23A mRNA expression level relative to patients age. Significance was calculated using Mann–Whitney test. * *p* < 0.05, ** *p* < 0.01, *** *p* < 0.001, **** *p* < 0.0001, and ‘ns’ indicates no significance.

To substantiate our initial findings from our cohort (Figure 1), the large-scale METABRIC dataset, which includes microarray expression from 1904 patients with invasive breast cancer was also explored. The clinicopathological parameters are described in Table 2. Our results showed that IL-23 was significantly linked to high-grade tumors compared to grade I (*p* < 0.0001) and grade II (*p* < 0.0001) (Figure 2a). This dataset includes an additional subgroup called Claudin-Low, which represents an aggressive subtype, characterized by the low expression of cellular junction proteins, and activation of the epithelial–mesenchymal transition (EMT) process [18]. In accordance with our experimental cohort, the data showed a significant association of IL-23 with aggressive molecular subtypes (Figure 2b) such as Claudin-Low, (Claudin-Low vs. LumA: *p* < 0.0001), (Claudin-Low vs. LumB: *p* < 0.0001), TNBC (TNBC vs. LumA: *p* < 0.0001), (TNBC vs. LumB: *p* < 0.0001) and HER2+ (HER2+ vs. LumA: *p* = 0.0001). Furthermore, IL-23 mRNA levels were elevated in tumors with negative hormonal receptors, ER− (*p* < 0.0001) and PR− (*p* < 0.0001) (Figure 2c,d), whereas no significant association with HER2 status was observed (Figure 2e). IL-23 levels were significantly higher in patients under 51 years old (*p* = 0.0098) and in those with a high Ki67 proliferation index (*p* < 0.0001) (Figure 2f,h).

In breast cancer, prognostic models facilitate clinical decision-making. Among the established tools is the Nottingham Prognostic Index (NPI), which was originally devised by Galea and colleagues. The NPI calculates a prognostic score based on tumor size, nodal status, and tumor grade [19]. This index predicts the clinical outcome of patients (prediction of 10-year survival after surgery). Stratification of the cohort into 4 prognostic groups using this prognostic index revealed that IL-23 expression was significantly linked to patients with moderate to poor survival predictions. Patients presenting poor and moderate scores had significantly elevated IL-23 expression compared to patients with excellent and good prognostic scores (Poor vs. Excellent (*p* = 0.0068); Poor vs. Good (*p* = 0.0003); (Moderate vs. Excellent (*p* = 0.0106); Moderate vs. Good (*p* < 0.0001)) (Figure 2g). Taken together, these results reveal a clear association between high levels of IL-23 and aggressive breast cancer behavior, characterized by adverse unfavorable clinicopathological features.

### 3.2. IL-23 Expression Correlated with Immunosuppressive Mediators in the TME

**Figure 3:**
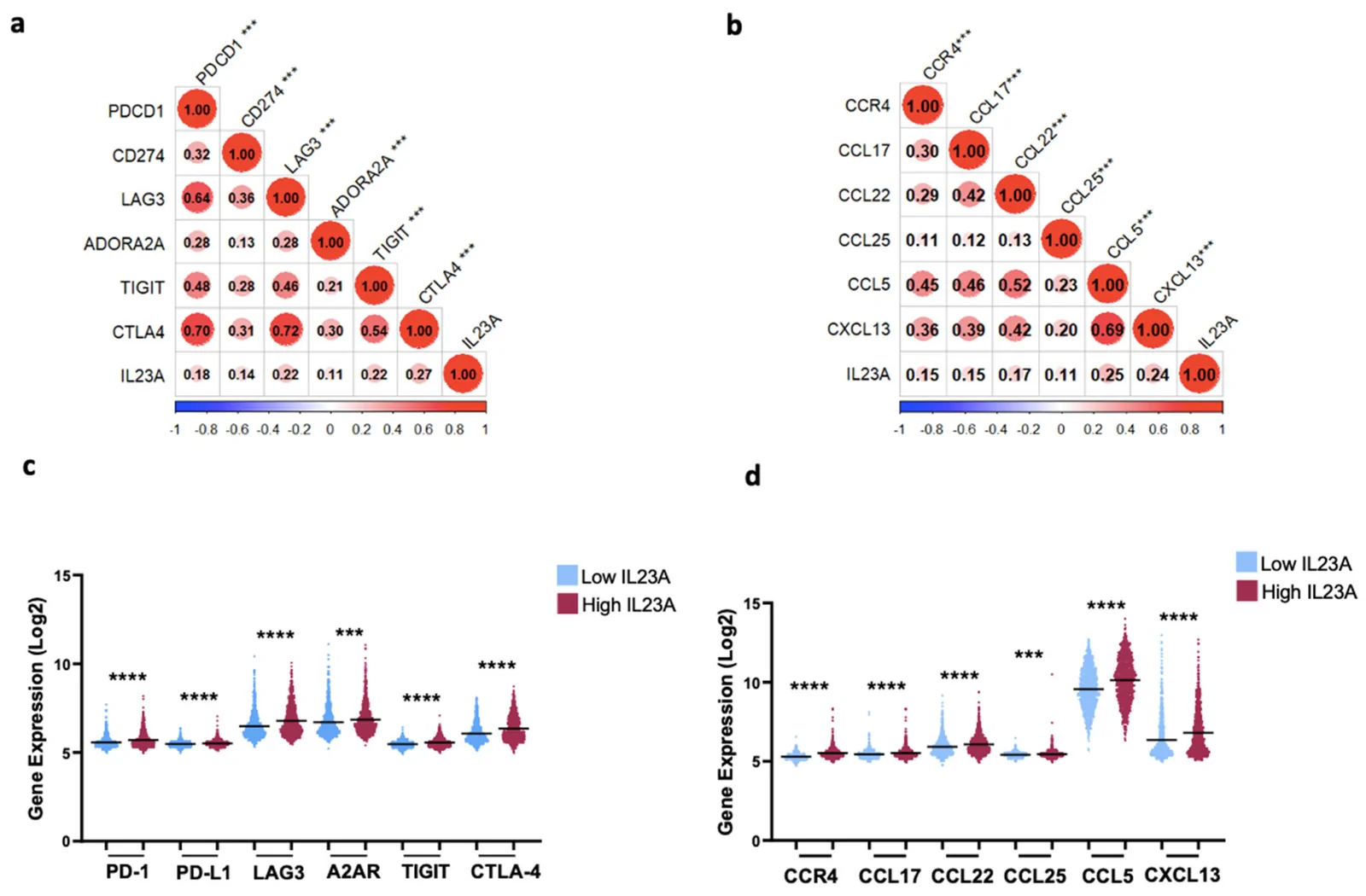
IL-23 was positively correlated with immunosuppressive molecular mediators in the METABRIC cohort. (**a**,**b**) Correlation analysis of IL-23 with inhibitory immune checkpoints and immunosuppressive chemokines. (**c**,**d**) Inhibitory immune checkpoint expression and immunosuppressive chemokines in High vs. Low IL-23 profiles (*n* = 1904). Statistical difference was calculated using the Mann–Whitney rank test. Spearman’s test was used for correlation coefficients. *** *p* < 0.001, **** *p* < 0.0001.

To explore the role of IL-23 in shaping the TME, we analyzed its correlation with the crucial molecular mediators involved in immunosuppression and tumor progression. We assessed the correlation of IL-23 with multiple inhibitory immune checkpoints (Figure 3a,c) and chemokines (Figure 3b,d), inducing attraction and polarization of tolerogenic and pro-tumoral cell subsets. Our results show that IL-23 was associated and positively correlated with immune checkpoints PDCD1 (PD-1), CD274 (PD-L1), LAG3, ADORA2A (A_2a_R), TIGIT and CTLA4, as well as chemokines CCR4, CCL17, CCL22, CCL25, CCL5 and CXCL13. These findings suggest that IL-23 may be associated with an immunosuppressive TME, potentially through its correlation with mediators involved in immune tolerance and tumor progression.

### 3.3. Virtual Screening Revealed IL-23RI as a Potential Inhibitor of IL-23 Receptor Binding

Based on the correlation results between IL-23 and immunosuppressive mediators, we next sought to investigate a potential strategy to reverse this immunosuppression by targeting the central IL-23/IL-17 axis. We hypothesized that direct inhibition of this pathway would effectively remodulate the TME. To identify a novel inhibitor of the IL-23/IL-23R interaction, we conducted high-throuput virtual screening, using the Maestro Schrödinger Suite. The protein preparation process for the structure (PDB ID: 5MZV) resulted in a refined model, ensuring structural integrity and accuracy. All identified *Allium sativum* L. compounds (Table S1, Supplementary Data) were retrieved in 3D SDF format for virtual screening. Molecular docking was carried out using the Glide module, where ligands were scored and ranked with the Extra Precision (XP) mode. The binding affinities of the top-ranked compounds were subsequently refined using the MM-GBSA method. From this analysis, a small molecule designated IL-23RI emerged as the top candidate, demonstrating the most favorable negative docking score of −7.482 kcal/mol and an MM-GBSA binding free energy of −48.80 kcal/mol, suggesting a stable binding interaction (Table 3). Furthermore, IL-23RI formed five hydrogen bonds with key residues: GLY 24, ASN 27, ASN 29, TYR 100 and GLU 111, located at the predicted interaction interface between IL-23 and its receptor. Based on these computational findings, IL-23RI was selected as a putative inhibitor of the IL-23/IL-23R complex for further experimental validation.

### 3.4. IL-23RI Specifically Interfered and Inhibited the IL-23/IL-17 Axis in Human T Cells

**Figure 4:**
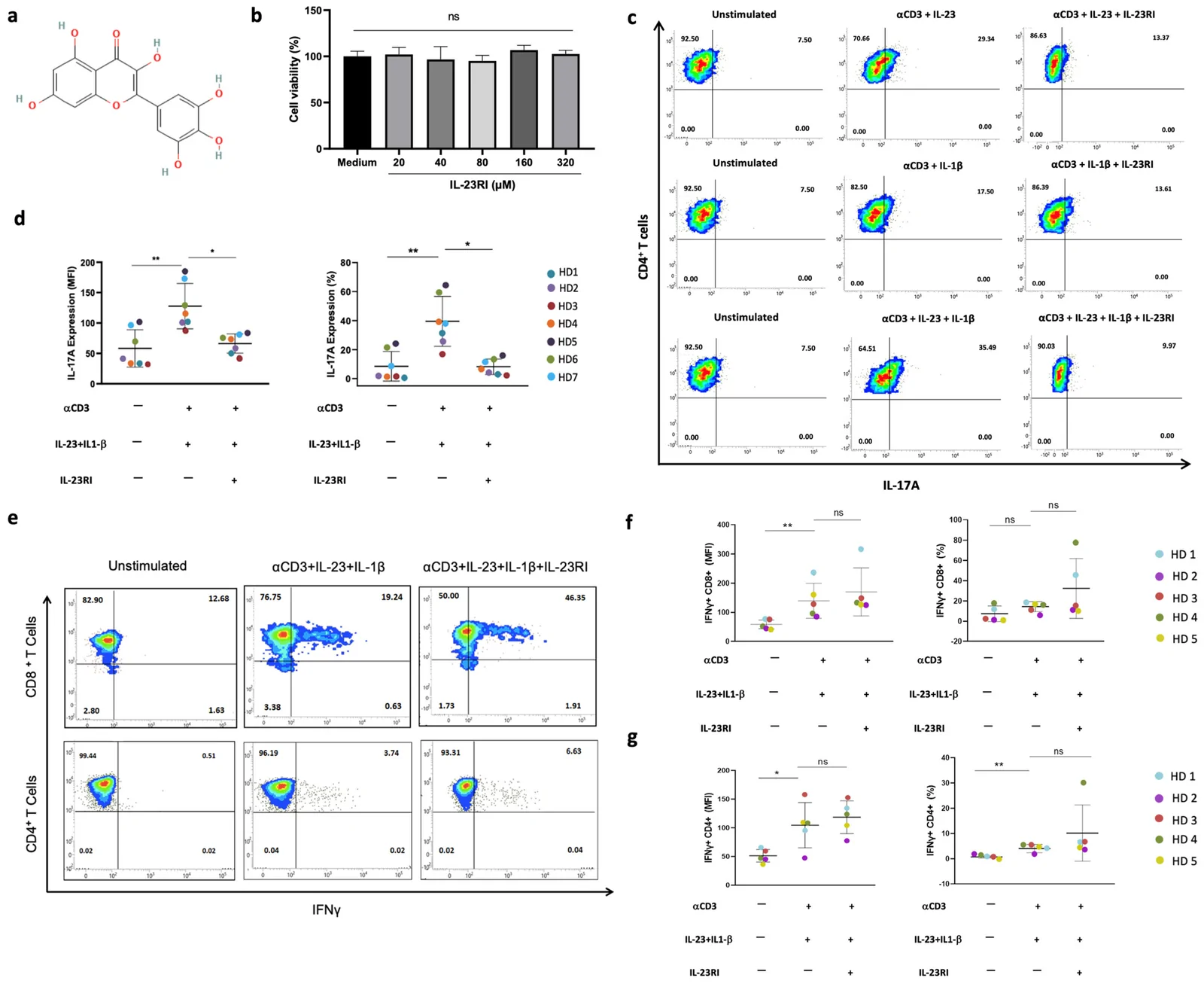
IL-23R blockade selectively inhibited IL-17A production by CD4^+^ T cells without affecting IFNγ production by CD8^+^ and CD4^+^ T cells. (**a**) IL-23RI chemical structure. (**b**) IL-23RI effect on healthy donor PBMCs viability at different concentrations (20–320 μM). Data are representative of 3 biological replicates. (**c**) Representative FACS plots of IL-17A^+^ CD4^+^ T cells under indicated conditions (160 µM IL-23RI). Specificity controls are shown from one representative donor of three; the inhibitory effect in the presence of both IL-23 and IL-1β was confirmed in seven donors. (**d**) Cumulative data showing IL-17A-producing cells under the indicated conditions, each point represents a unique donor (*n* = 7). (**e**) The data show representative FACS plot of IL-23RI effect (160 µM) on IFNγ secretion by CD4^+^ and CD8^+^ T cells. Data are representative of 5 biological replicates. (**f**) IFNγ-producing CD8^+^ T cells under the indicated conditions, each point represents a unique donor. (**g**) Cumulative data showing IFNγ-producing CD4^+^ T cells under the indicated conditions, each point represents a unique donor. Data are represented as mean ± SD. * *p* < 0.05; ** *p* < 0.01; ns = not significant ((**b**) Kruskal–Wallis test was applied followed by Dunn’s multiple comparisons; (**d**,**f**,**g**) Friedman followed with Dunn’s post hoc was applied).

Following the identification of IL-23RI as a candidate IL-23R inhibitor (Figure 4a), we next evaluated its biological activity. We first assessed its effect on human PBMC viability using an MTT assay. As shown in Figure 4b, IL-23RI did not induce significant cytotoxicity, even at concentrations up to 320 µM. Before assessing the capacity of IL-23RI to inhibit the IL-23/IL-17 axis, we first performed control experiments to verify the specificity of the compound. PBMCs from three healthy donors were stimulated with anti-CD3 in the presence of recombinant human IL-23 alone, IL-1β alone, or both cytokines, with or without IL-23RI (160 µM). After five days, IL-17A secretion by CD4^+^ T cells was measured in each condition. Representative flow cytometry analysis from one donor (Figure 4c) showed that IL-23RI does not considerably reduce Il-17A production in the presence of IL-1β alone (13.61% vs. 17.50% in the stimulated condition, compared to 7.50% in the unstimulated cells). In contrast, IL-23RI markedly inhibited IL-17A production in the presence of IL-23 alone (13.37% vs. 29.34% in the stimulated condition, baseline 7.50%). Moreover, in the presence of both cytokines, IL-23RI potently reduced IL-17A production (9.97% vs. 35.49% in the stimulated condition, baseline 7.50%). These results suggest that IL-23RI exerts an inhibitory effect primarily in the presence of IL-23, indicating a potential selectivity for the IL-23/IL-17 axis. This effect was reproducible across the three donors. To further validate this observation, we extended the analysis to four additional healthy donors, for a total of seven donors, under the three conditions: unstimulated, stimulated with anti-CD3 + IL-23 + IL-1β, and stimulated with anti-CD3 + IL-23 + IL-1β + IL-23RI. Cumulative data from all seven donors (Figure 4d) confirmed a significant inhibition of IL-17A secretion, which was consistent whether measured by the percentage of IL-17A^+^ CD4^+^ T cells (*p* = 0.0458) or by Mean Fluorescence Intensity (MFI) (*p* = 0.0458) (Figure 4d).

We further investigated whether IL-23RI could modulate T-cell function by affecting IFNγ production. PBMCs from five healthy donors were cultured and stimulated as described above (Figure 4d) using the same three conditions: unstimulated, stimulated with anti-CD3 + IL-23 + IL-1β, and stimulated with anti-CD3 + IL-23 + IL-1β + IL-23RI (160 µM). Flow cytometry analysis after overnight incubation suggested a trend toward increased IFNγ production, particularly in CD8^+^T cells. In a representative donor, the frequency of IFNγ^+^CD8^+^ T cells increased from 19.24% in the stimulated cells to 46.35% following IL-23RI treatment compared to a baseline of 12.68% in the unstimulated cells (Figure 4e). Similarly, the frequency of IFNγ^+^CD4^+^ T cells increased from 3.74% to 6.63%, from a baseline of 0.51% (Figure 4e). However, cumulative data from five donors demonstrated that this apparent increase was not statistically significant whether measured by cell frequency (*p* = 0.8413 for IFNγ^+^CD8^+^; *p* = 0.3095 for IFNγ^+^CD4^+^T) or by MFI (*p* = 0.6429 for IFNγ^+^CD8^+^; *p* = 0.6508 for IFNγ^+^CD4^+^T) (Figure 4f,g). Collectively, these findings suggest that IL-23RI selectively inhibits the IL-23/IL-17 axis without inducing a broad enhancement of IFNγ-producing T cells.

### 3.5. IL-23RI Impaired MCF-7 Breast Cancer Cell Migration In Vitro

**Figure 5:**
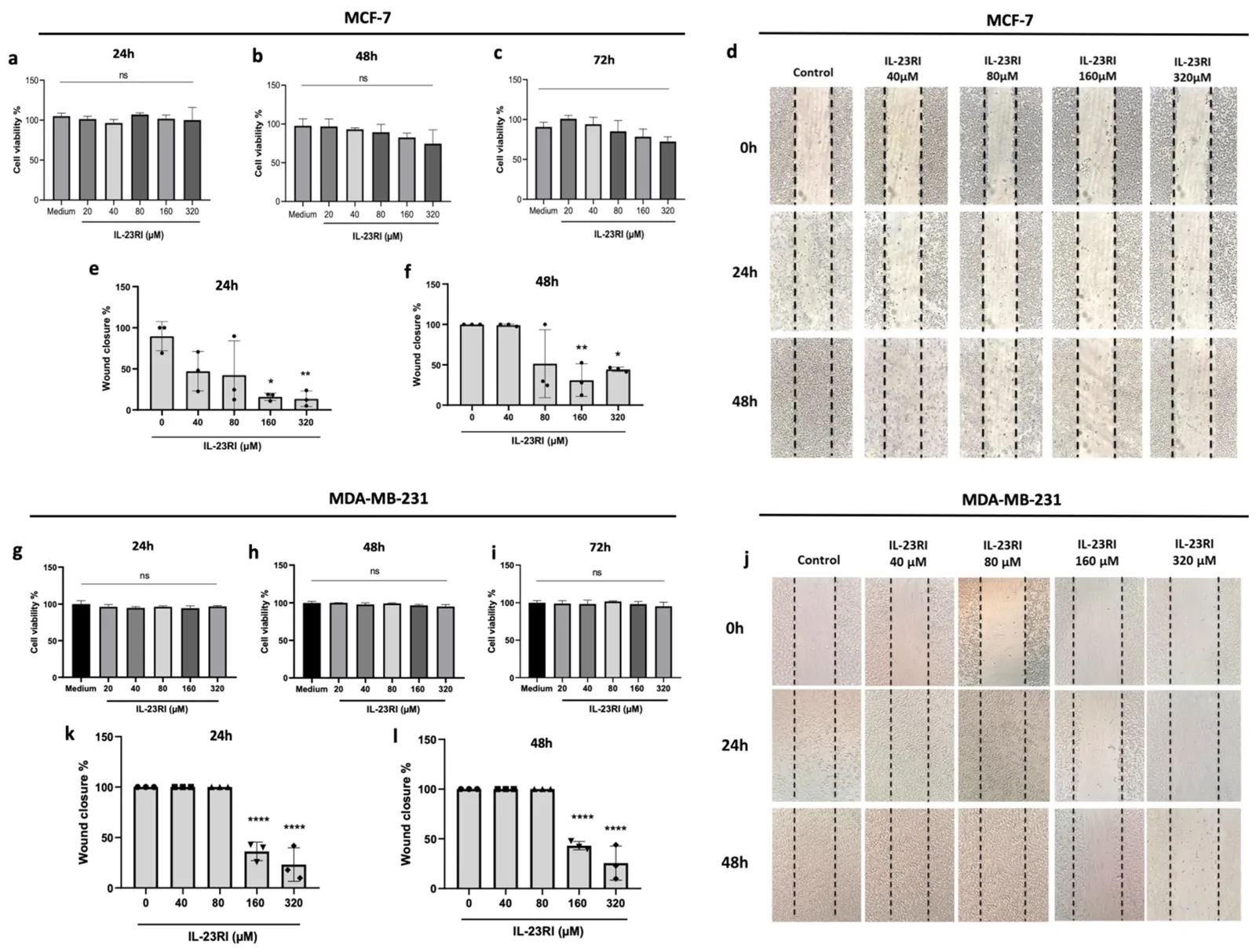
Anti-migratory effect of IL-23RI on breast cancer cells. (**a**–**c**,**g**–**i**) Evaluation of IL-23RI effect on MCF-7 and MDA-MB-231 human breast cancer cell viability in the absence (Medium), or in the presence of indicated concentrations at 24 h, 48 h and 72 h. Data are representative of 3 biological replicates. (**d**,**j**) The effect of IL23-RI on MCF-7 and MDA-MB-231 cells migration evaluated by wound-healing assay for 24 and 48 h post-treatment with indicated concentrations. Data are representative of 3 biological replicates. (**e**,**f**,**k**,**l**) The percentage of wound closure relative to untreated control at 24 and 48 h, respectively for each cell line. Data are expressed as mean ± SD of 3 biological replicates. * *p* < 0.05; ** *p* < 0.01; **** *p* < 0.0001; ns = not significant. One-way ANOVA followed by Dunnett’s multiple comparisons test was applied.

To explore the anti-cancer potential of IL-23RI, we first assessed its direct cytotoxicity on MCF-7 breast cancer cells. An MTT assay confirmed that IL-23RI exhibited no cytotoxic effects at any tested concentration or time point (24, 48 and 72 h) (Figure 5a–c). We then investigated its impact on cancer cell migration using a wound-healing assay. IL-23RI demonstrated a potent dose-dependent anti-migratory effect (Figure 5d). Treatment with IL-23RI at 160 µM and 320 µM significantly inhibited wound closure (*p* = 0.0102 for 160 µM; *p* = 0.0084 for 320 µM), yielding rates of 18.79% and 12.25% at 24 h (Figure 5e), and 52.50% and 46.55% at 48 h (*p* = 0.0079 for 160 µM; *p* = 0.0277 for 320 µM) (Figure 5f), respectively. This is in stark contrast to the untreated control, which achieved 69.21% closure at 24 h and 100% at 48 h. The 40 µM concentration showed moderate inhibition at 24 h (22.74% closure), but the wound was nearly closed by 48 h (97.52%). The data for the 80 µM concentration was inconsistent and not reproducible across the three independent experiments. These results indicate that IL-23RI exerts anti-migratory effects on MCF-7 cells without inducing cytotoxicity.

To further validate these findings in a more aggressive breast cancer model, we extended our analysis to MDA-MB-231 cells, a highly invasive triple-negative breast cancer (TNBC) cell line. As for MCF-7, we first assessed the effect of IL-23RI on cell viability. An MTT assay confirmed that IL-23RI did not induce cytotoxicity at any tested concentration or time point (Figure 5g–i). We then performed wound-healing assays to evaluate its effect on MDA-MB-231 cell migration. Treatment with IL-23RI at 160 µM and 320 µM significantly inhibited wound closure (*p* < 0.0001 for 160 µM; *p* < 0.0001 for 320 µM), yielding rates of 42.80% and 17.862% at 24 h (Figure 5k), and 47.76% and 22.96% at 48 h (*p* < 0.0001 for 160 µM; *p* < 0.0001 for 320 µM) (Figure 5i), respectively. In contrast, the untreated control, as well as the 40 µM and 80 µM conditions, achieved 100% closure at both 24 h and 48 h. Collectively, these findings suggest that IL-23RI exerts anti-migratory effects on both MCF-7 and MDA-MB-231 breast cancer cells, without inducing cytotoxicity.

## 4. Discussion

The TME reflects a complex and dynamic network of cellular and molecular interactions that dictates tumor progression and therapeutic response [20,21]. In breast cancer, a profoundly immunosuppressive profile facilitates immune escape, a process driven by inhibitory immune checkpoints and soluble mediators such as cytokines [22,23]. Chronic inflammation is a well-established driver of tumorigenesis, metastasis and therapy resistance [7,8,24]. Our study focused on the pro-inflammatory cytokines, especially IL-23, a key mediator of chronic inflammation, exploring its role in breast cancer aggressiveness and introducing IL-23RI, a small natural compound, as a promising therapeutic agent capable of disrupting the IL-23/IL-17 axis.

The first part of this work identified IL-23 as a potential marker of aggressive breast cancer. Transcript analysis in a Moroccan cohort revealed that IL-23 mRNA is overexpressed in tumors with negative estrogen and progesterone status as well as in HER2+ and TNBC molecular subtypes. Validation using the large-scale METABRIC cohort corroborated these results, showing a significant association between high IL-23 levels and high tumor grade, aggressive molecular subtypes (including Claudin-low), and poor survival as defined by the Nottingham Prognostic Index. These observations are consistent with a previous study by Shuhai Sheng and colleagues, who reported that elevated IL-23 and its receptor IL-23R in breast tumor tissues are linked to larger tumor size, advanced TNM stage, and metastasis [13]. In addition, increased IL-23 levels have been associated with colon and rectal adenocarcinoma [25]. Together, this cumulative evidence supports the potential role of IL-23 as an indicator of poor prognosis.

The immunosuppressive TME releases several molecular factors that may polarize cells toward a pro-tumoral phenotype, which has been implicated in tumor aggressiveness [4,11]. Our results show that IL-23 expression is positively correlated with a range of inhibitory immune checkpoints, including PDCD1 (PD-1), CD274 (PD-L1), LAG3, ADORA2A (A2AR), TIGIT and CTLA4, as well as with immunosuppressive chemokines such as CCR4, CCL17, CCL22, CCL25, CCL5 and CXCL13. Thus, IL-23 was found to be positively and significantly correlated and associated with these mediators of immune evasion. These findings suggest a potential link between IL-23 and the immunosuppressive TME.

Within the TME, IL-23 abundance induces the upregulation of its own receptor, IL-23R [26]. The binding of IL-23 to its receptor activates a signaling pathway that drives the differentiation of IL-17A-producing CD4^+^T helper (Th17) cells via the key transcription factors STAT3 and RORγt [27]. This pathway promotes tumor progression through multiple mechanisms. Previous studies have indicated that IL-23 and IL-23R can promote tumor growth and may decrease immunosurveillance by CD8^+^ T cells in several types of cancers, including skin, melanoma and breast cancers [28]. In mammary cancer, the tumor-promoting effect of IL-23 was mediated by the recruitment and infiltration of immunosuppressive M2 macrophages and neutrophils in the TME [22]. Furthermore, IL-17A expression was correlated with increased tumor microvascular density, enhanced metastasis and an unfavorable prognosis [29]. Luciana Benevides and colleagues demonstrated that IL-17A-producing cells are positively correlated with the presence of regulatory T cells (Tregs), tumor aggressiveness and poor prognosis in mammary tumor models [30]. Collectively, these findings suggest that the IL-23/IL-17 axis may play a role in the immunosuppressive TME and to processes linked with metastasis, offering a rationale for further exploring this pathway as a therapeutic target.

To this end, a high-throuput virtual screening was employed to identify a potential novel small natural inhibitor targeting the IL-23/IL-23R interaction. Among the screened compounds, IL-23RI was selected based on its predicted binding affinity to the IL-23R pocket. We therefore performed in vitro experiments to assess its functional effects. Notably, and without any detectable cytotoxicity, IL-23RI significantly reduced IL-17A production by CD4^+^ T cells under the combined stimulation conditions (anti-CD3 + IL23 + IL-1β + IL-23RI). In contrast, IL-23RI did not markedly affect IL-17A production in the absence of IL-23. The reproducibility of the control experiments across three donors suggests that IL-23RI may act specifically on the IL-23/IL-17A axis, potentially through IL-23R binding. No significant increase in IFNγ was observed, suggesting that IL-23RI does not appear to broadly activate T cells. The slight, non-significant trend toward increased IFNγ may be due to the limited sample size and donor variability, warranting further investigation. Together, these findings suggest that IL-23RI primarily targets the IL-23/IL-17 inflammatory signaling axis rather than inducing a general T-cell activation. This selective inhibition may represent a favorable feature for an immunomodulatory candidate, as it could potentially avoid non-specific immune activation.

Beyond its immunomodulatory properties, IL-23RI exhibited a direct anti-migratory effect on both MCF-7 and MDA-MB-231, the latter representing an aggressive TNBC subtype. Notably, this effect suggests an additional property of the compound. The inhibition of migration at non-cytotoxic concentrations supports a potential dual functionality, although further experiments are needed to determine whether this effect is mediated through IL-23R or through a distinct target. The selectivity of IL-23RI is particularly noteworthy; while it significantly reduced cell motility, it did not affect cell viability, suggesting that IL-23RI may preferentially interfere with cellular processes involved in migration, a key step in the metastatic cascade. These findings extend the potential relevance of IL-23RI beyond its immunomodulatory activity and warrant further investigation into its direct effects on tumor cell behavior.

In conclusion, our study identifies IL-23 as a potential driver of breast cancer aggressiveness and suggests an association with an immunosuppressive signature, although functional validation using immunohistochemistry is still required. To our knowledge, this study is the first to introduce a small natural molecule, IL-23RI, as a candidate therapeutic agent targeting the pro-inflammatory IL-23/IL-17 axis in breast cancer. Its dual functionality, modulating the IL-23/IL-17 axis and inhibiting tumor cell migration, positions IL-23RI as a promising candidate for further development. However, the direct binding of IL-23RI to the IL-23 receptor has been predicted by molecular docking but awaits confirmation using biophysical methods such as Surface Plasmon Resonance (SPR) or Isothermal Titration Calorimetry (ITC). We also acknowledge that the concentrations used in our functional assays (160 µM for PBMC assays and 160–320 µM for migration assays) are relatively high for a small natural molecule, and further optimization, including a full dose–response analysis to determine the IC_50_ will be required. In addition, further studies with larger cohorts are needed to fully elucidate the mechanistic involvement of IL-23R in the observed effects. Future detailed studies using IL-23R knockdown (siRNA) or neutralizing antibodies, as well as analysis of downstream signaling components such as STAT3 phosphorylation and RORγt expression, will be required to confirm pathway specificity. Finally, in vivo validation using preclinical breast cancer models will be necessary to assess the therapeutic potential of IL-23RI and to investigate its impact on the tumor microenvironment, including various T-cell subsets.

## Figures and Tables

**Table 1:** IL-23 gene expression across different clinicopathological features in the Moroccan cohort.

Clinicopathological Parameters	Cases (%) (*n* = 34)	*p*-Value
Age		
▪ <51 ▪ ≥51 ▪ Missing data	15 (44.11%) 15 (44.11%) 4 (11.76%)	0.3669
Histological grade		
▪ I ▪ II ▪ III	3 (8.82%) 13 (38.24%) 18 (52.94%)	0.1282
Molecular subtype		
▪ Luminal A ▪ Luminal B ▪ HER2 ▪ TNBC	8 (23.53%) 9 (26.47%) 7 (20.59%) 10 (29.41%)	0.0033
ER status		
▪ ER+ ▪ ER− ▪ Missing data	15 (44.12%) 16 (47.06%) 3 (8.82%)	0.0011
PR status		
▪ PR+ ▪ PR− ▪ Missing data	16 (47.06%) 17 (50%) 1(2.94%)	0.0059
HER2 status		
▪ HER2+ ▪ HER2− ▪ Missing data	10 (29.41%) 23 (67.64%) 1(2.94%)	0.5499
Ki-67 proliferation index		
▪ Low-Ki-67 ▪ High-Ki-67 ▪ Missing data	9 (26.47%) 16 (47.06%) 9 (26.47%)	0.5499

HER2: human epidermal growth factor receptor-2, TNBC: triple-negative breast cancer, ER: estrogen receptor and PR: progesterone receptor. Statistical comparison were performed using Mann–Whitney (two groups) or Kruskal–Wallis test (multiple groups).

**Table 2:** IL-23 gene expression across different clinicopathological features in METABRIC cohort.

Clinicopathological Parameters	Cases (%) (*n* = 1904)	*p*-Value
Age		
▪ <51 ▪ ≥51	411 (21.59%) 1493 (78.41%)	0.0098
Histological grade		
▪ G1 ▪ G2 ▪ G3 ▪ Missing data	165 (8.66%) 740 (38.86%) 927 (48.68%) 72 (3.78%)	<0.0001
Molecular Subtype		
▪ Luminal A ▪ Luminal B ▪ HER2 ▪ TNBC ▪ Claudine-low ▪ Missing data	679 (35.66%) 461 (24.21%) 220 (11.55%) 199 (10.45%) 199 (10.45%) 146 (7.67%)	<0.0001
PR status		
▪ PR+ ▪ PR−	1009 (52.99%) 895 (47.01%)	<0.0001
ER status		
▪ ER+ ▪ ER−	1459 (76.63%) 445 (23.37%)	<0.0001
HER2 status		
▪ HER2+ ▪ HER2−	236 (12.39%) 1668 (87.60%)	0.0678
Ki-67 proliferation index		
▪ Low-Ki-67 ▪ High-Ki-67	1073 (56.35%) 831 (43.64%)	<0.0001
NPI score category		
▪ Excellent (≤2.4) ▪ Good (2.4–3.4) ▪ Moderate (3.4–5.4) ▪ Poor (>5.4)	163 (8.56%) 477 (25.05%) 1070 (56.20%) 194 (10.18%)	<0.0001

HER2: human epidermal growth factor receptor-2, TNBC: triple-negative breast cancer, ER: estrogen receptor and PR: progesterone receptor. Statistical comparisons were performed using Mann–Whitney (two groups) or Kruskal–Wallis test (multiple groups).

**Table 3:** Molecular docking results and interaction profiles of the screened ligands.

Ligand	Docking Score (Kcal/mol)	MM-GBSA ΔG Bind (Kcal/mol)	Residues of Interaction
IL-23RI	−7,482	−48.80	GLY24, ASN27, ASN29, TYR100 and GLU111
Quercetin	−6.117	−40.21	GLY24, ASN27, ASN29 and TYR100
Cianidanol	−4.425	−38.19	TYR100, GLU111 and LEU113

## Data Availability

The original contributions presented in this study are included in the article/Supplementary Material. Further inquiries can be directed to the corresponding author(s).

## Supplementary Materials

The following supporting information can be downloaded at: https://www.mdpi.com/article/10.3390/medsci14040485/s1, Table S1. Chemical components identified in *Allium sativum* L. [31,32,33,34,35,36,37,38].

## Abbreviations

TME: Tumor microenvironment; Lum A: Luminal A; Lum B: Luminal B; TNBC: Triple-negative breast cancer; HER2: Human epidermal growth factor receptor 2; IL-23: Interleukine 23; IL-23R: Interleukine 23 receptor; IL-17: Interleukine 17; IL-23RI: Interleukine 23 receptor inhibitor; TGFβ: Transforming growth factor β; Th17: T helper 17 cells; RORγT: RAR-related orphan receptor gamma t; ER: Estrogen receptor; PR: Progesterone receptor; METABRIC: Molecular taxonomy of breast cancer international consortium; NPI: Nottingham prognosis index; PD-1: Programmed cell death protein 1; PD-L1: Programmed death ligand 1; LAG3: Lymphocyte-activation gene 3; A_2a_R: Adenosine receptor 2a; TIGIT: T-cell immunoreceptor with Ig and ITIM domains, CTLA-4: Cytotoxic T-lymphocyte associated protein 4; CCR4: CC chemokine receptor 4; CCL17: C-C motif chemokine ligand 17; CXCL13: C-X-C motif chemokine ligand 13; PBMC: Peripheral blood mononuclear cells; IFNγ: Interferon gamma; MM-GBSA: Molecular mechanics/Generalized born surface area.
