# Supplementary material for: A Small Natural Molecule Targeting IL-23/IL-17 Axis Exerts Dual Effects on T-Cell Function and Breast Cancer Cell Migration
Source: Med Sci (Basel). 2026 Aug 15;14(4):485. doi: 10.3390/medsci14040485 (PMC13515054; doi:10.3390/medsci14040485)
Supplement: Supplementary file 1 [file medsci-14-00485-s001.zip › medsci-4356941-supplementary.pdf]

Supplementary Information.

Table S1. Chemical components identified in *Allium sativum* L.

Components	References
Allicin ; Methyl allylthiosulfinate ; 1-propenyl allylthiosulfinate; Allylmethylthiosulfinate (E) Ajoene ; (Z)-Ajoene ; S-ALLYL-D-Cysteine; Gamma-Glutamylcysteine; S-allyl cysteine sulfoxide; Selenomethionine ; selenocystathionine ; Se-cystine Allyl Disulfide ; Propyl disulfide; Methyl Disulfide; 3-vinyl-6H-1,2-dithiin; 3-vinyl-6H-1,3-dithiin ; Ethyl vinyl sulfide ;Methyl propyl Disulfide; Methyl allyl Disulfide; Dimethyl trisulfide; Butyl methyl trisulfide; Diallyltrisulfide; Diallyl Sulfide	[31]
Alliin ; L-Deoxyalliin ; S-allyl-L-cysteine; 1,2-Dithiacyclopentane; methyl ester; Benzyl sulfanylacetic acid; S-allyl mercapto-L-cysteine; gamma-L-Glutamyl-S-(2-carboxy-1 propyl) cysteinylglycine ; y-L-Glutamyl-L-methionine; y-L-Glutamyl-S-methyl-L cysteinesulfoxide ; y-L-Glutamyl-S-allylthio-L-cysteine; y-L-Glutamyl-S-methyl-L-cysteine ; S (2-carboxy-n-propyl)-glutathione ; y-L-Glutamyl-S-allyl-L-cysteine ; y-L-Glutamyl-L phenylalanine ; y-L-Glutamyl-L-isoleucine ; y-L-Glutamyl-L-leucine ; y-L-Glutamyl-L-valine ; y-L-Glutamyl-S-(l-propenyl)-cysteine-S-oxide ; y-L-Glutamyl-S-propylcysteine ; S propylcysteinesulfoxide ; Methylcysteine ; S-Allyl-L-cysteine ; γ-glu-SeMeSeCys ; γ-glutamyl Semethylselenomethionine; γ-glutamyl-Se-methionine; γ-glutamyl Se-methylselenocysteine ; Methional ;Carvone ; 1,4-dimethyl tetrasulfide (DMTTS) ; Proto-eruboside B; proto-iso eruboside B; eruboside B; iso-eruboside B; sativoside B1; Pro-desgalactotigonin; Sativoside R1; Sativoside R2; voghieroside A1 ; voghieroside A2 ; voghieroside B1 ; voghieroside B2 ; voghieroside C1 ; voghieroside C2 ; voghieroside D1 ; voghieroside D2 ; voghieroside E1 ; voghieroside E2 ; Allyl methyl sulfide ; S-Methylcysteinesulfoxide; Allyl 2,3 epoxypropylsulfide;	[32]
Quercetin, Myricetin, Apigenin, β-Sitosterol, Campesterol, Stigmasterol, Kaempferol, 1-Octadecanol.	[33,34]
MethylAllylTrisulfide; 3-Vinyl-4H-1,2-dithiin; 2-Vinyl-4H-1,3-dithiin; Pentadec-1-ene; Diallyltetrasulfide; Sodium ; 1,2,3,4-tetrathia-5-methyl cyclohexane; 3-(2-thia-4-pentenyl)-1 thiacyclohex-5-ene; 3-(2,3,4-trithia-5-heptenyl-1-thia-cyclohex-5-ene); 2-(1-thia-2-cyclohexen 6-yl)-1,3-dithia-cyclohex-5-ene ;2-Amino-4,4,6,6-tetramethyl-4,6-dihydrothieno-[2,3-c]furan-3 carbonitrile ; 2-Trimethylsilyl-1,3-dithiane ; Spiro(17-acetoxyandrostan-3-ol)2.2'(1',3'-dithiane); 2-propenylthioacetonitrile; 3,5-Diethyl-1,2,4-trithiolane; 3,4-Dihydroxybenzoic Acid ; 3-methylthio propanal; Nicotinicacid.	[35]
Caffeic acid; ferulic acid ; Sinapic Acid; Vanillic acid; 4-Hydroxybenzoic Acid; Naringenin; Catechin; Gallocatechol; Rutin; Nobiletin; p-Coumaric Acid; Linalool.	[36]
2, 3-Dimethoxybenzoic acid; Epicatechin; Gallic acid; Syringic acid.	[37]
Thiacremonone; 2-Aminoethanethiolsulfuric acid.	[38]
